# Accelerated atherogenesis in completely ligated common carotid artery of apolipoprotein E-deficient mice

**DOI:** 10.18632/oncotarget.22685

**Published:** 2017-11-25

**Authors:** Zhihui Chang, Chaoji Huangfu, Andrew T. Grainger, Jingang Zhang, Qiyong Guo, Weibin Shi

**Affiliations:** ^1^ Department of Radiology & Medical Imaging, University of Virginia, Charlottesville, Virginia, USA; ^2^ Department of Radiology, Shengjing Hospital of China Medical University, Shenyang, China; ^3^ Beijing Key Laboratory of Blood Safety and Supply Technologies, Beijing Institute of Transfusion Medicine, Beijing, China; ^4^ Biochemistry & Molecular Genetics, University of Virginia, Charlottesville, Virginia, USA

**Keywords:** atherosclerosis, hyperlipidemia, neovessel, foam cell, mice

## Abstract

Complete ligation of the common carotid artery near its bifurcation induces neointimal formation due to smooth muscle cell proliferation in normolipidemic wild-type mice, but it was unknown what would happen to hyperlipidemic apolipoprotein E-deficient (Apoe^-/-^) mice. Examination of these mice revealed rapid development of atherosclerotic lesions in completely ligated carotid arteries within 4 weeks. Mice were fed a Western diet, starting 1 week before ligation, or a chow diet. Foam cell lesions formed as early as 1 week after ligation in mice fed the Western diet and 2 weeks in mice fed the chow diet. Fibrous lesions comprised of foam cells and smooth muscle cells and more advance lesions containing neovessels occurred at 2 and 4 weeks after ligation, respectively, in the Western diet group. Lesions were larger and more advanced in the Western diet group than the chow group. Neutrophil infiltration was observed in growing intimal lesions in both diet groups, while CD8+ T cells were found in lesions of chow-fed mice. This study demonstrates that Apoe^-/-^ mice develop the entire spectrum of atherosclerosis in ligated carotid arteries in an accelerated manner and this model could be a valuable tool for investigating the development and therapy of atherosclerosis.

## INTRODUCTION

Atherosclerosis is the primary cause of heart attack, ischemic stroke and peripheral arterial disease that kill more people than a single other disease in the Western countries [1]. Atherosclerotic lesions may directly or indirectly, through formation of thrombi, block the blood flow to such important organs as the heart and brain [2]. Angioplasty/stenting is a common and effective treatment to occlusive arterial diseases. However, a fraction of patients receiving the treatment develop significant restenosis, the reduction of the luminal size due to loss of gain in luminal size after angioplasty [2]. The placement of new-generation drug-eluting stents has dramatically reduced the incidence of post-interventional restenosis, down to ∼10% within a 5-year period [3]. Most stent failures are attributable to in-stent thrombosis, neointimal hyperplasia and/or recurrent atherosclerosis.

The mouse is widely used to elucidate the pathological basis of various human diseases due to the availability of numerous inbred strains, knockouts and transgenics [4]. Neointimal hyperplasia can be induced in the common carotid artery of FVB/NJ mice by ligating the vessel near its distal end [5]. Smooth muscle cells can be observed in the intima as early as 5 days after ligation, their replication rate reaches its peak at 2 weeks, and by 4 weeks, intimal lesion sizes stop growing. Subsequent studies, however, indicate that neointimal growth in the ligated common carotid artery is very limited in most wild-type mouse strains, including C57BL/6 (B6) and C3H/HeJ [6]. In contrast, when deficient in apolipoprotein E (Apoe^-/-^) and fed a high-fat diet, B6 mice developed prominent atherosclerotic lesions containing cholesterol clefts and intraplaque neovessels in partially ligated common carotid artery within 4 weeks [7]. However, no study has been carried out on Apoe^-/-^ mice under either a chow or high fat diet condition to investigate lesion formation in a completely ligated carotid artery. These mice develop spontaneous hyperlipidemia and atherosclerosis on a low-fat, low cholesterol chow diet [8][9], which is accelerated by feeding a high-fat diet [10]. The primary aim of this study was to characterize lesion formation following complete ligation of one common carotid artery in Apoe^-/-^ mice, including the types of cells present and the sequence of cellular events that occurred.

## RESULTS

### Plasma lipid levels of Apoe^-/-^ mice

**Figure 1:**
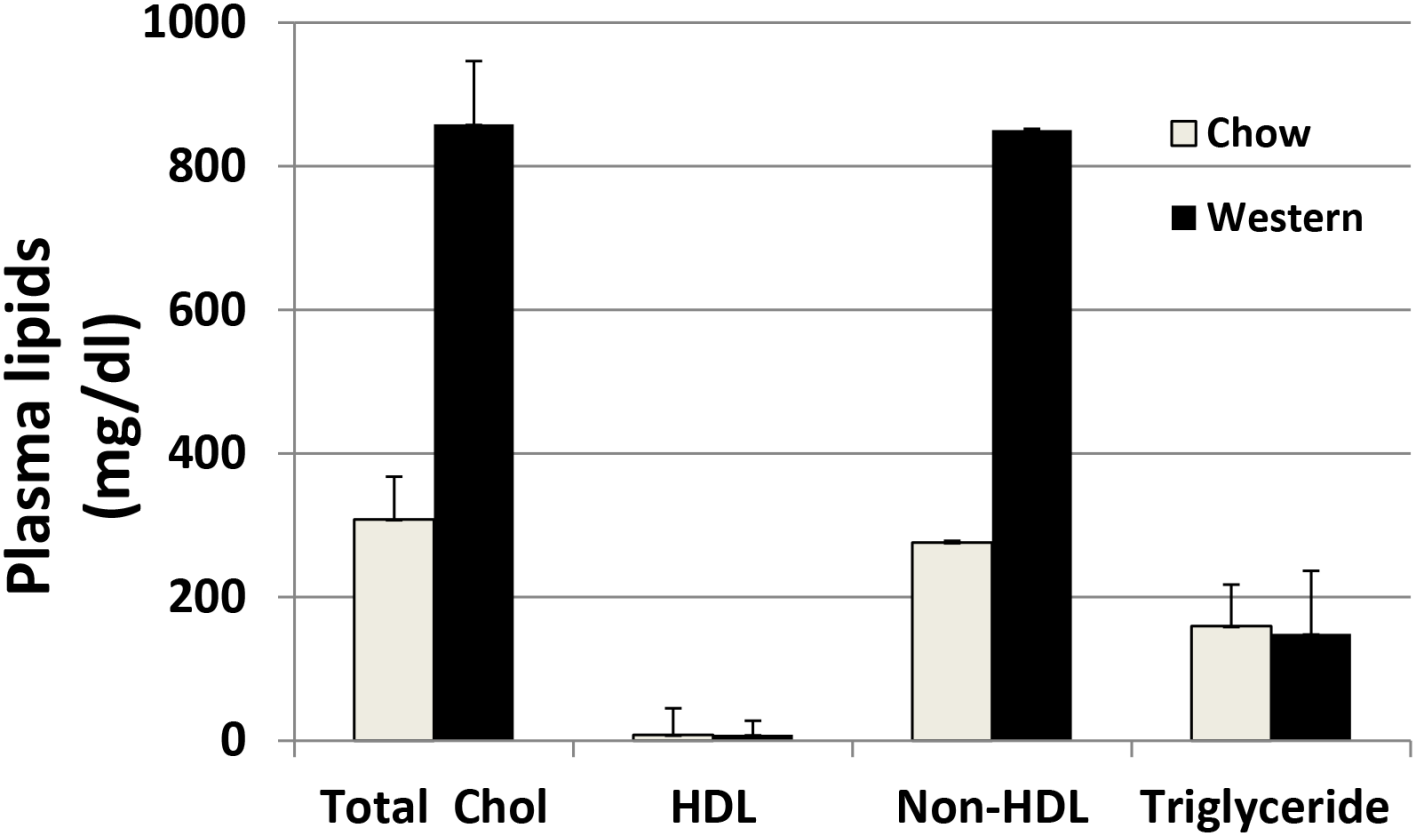
Plasma levels of total cholesterol (total chol), HDL cholesterol, and triglyceride in Apoe^−/−^ mice fed a chow diet and a Western diet Blood samples were obtained after mice were fasted overnight. Values are means ± SE for 3 or 5 male mice per group.

Plasma lipid levels were analyzed for Apoe^-/-^ mice fed with a chow diet and a Western diet (Figure 1). As expected, these mice developed mild hyperlipidemia with a total cholesterol level of 308 ± 60 mg/dl on the chow diet and severe hyperlipidemia with a total cholesterol level of 858 ± 88 mg/dl on the Western diet. The HDL cholesterol levels were low on either diet (8 ± 2 mg/dl). Triglyceride levels were comparable between the chow and Western diet groups (159 ± 37 vs. 149 ± 19 mg/dl).

### Lesion development

**Figure 2:**
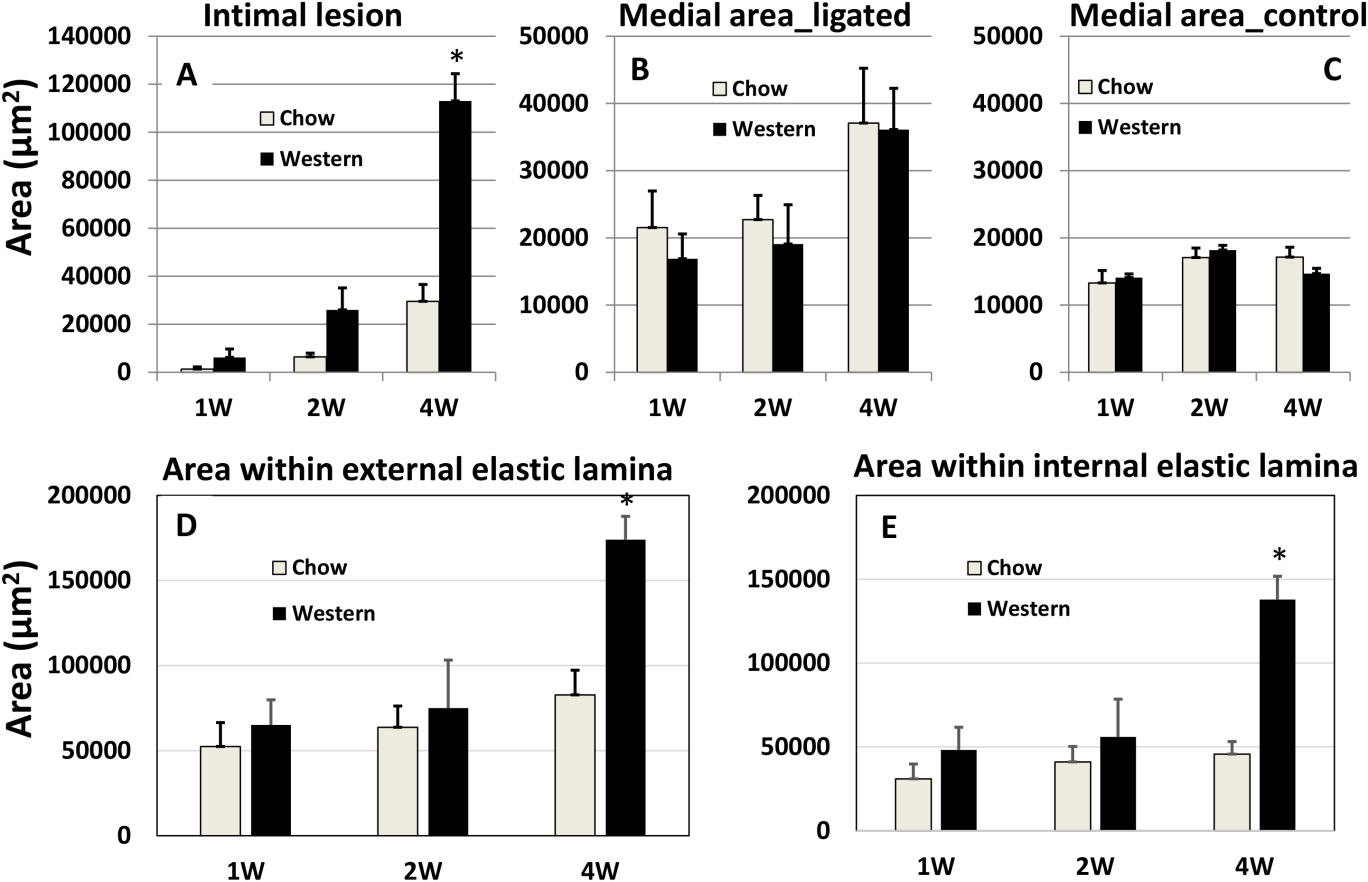
Quantitative measurements of intimal **(A)** and medial areas **(B)** in ligated left common carotid arteries and medial areas in the contralateral common carotid arteries **(C)** of Apoe^-/-^ mice fed with a chow diet and a Western diet. Cross-sectional areas encircled by the external elastic lamina **(D)** and internal elastic lamina **(E)** in the ligated arteries were also presented. Measurements were made on the carotid arteries 1, 2 and 4 weeks after ligation. Values are means ± SE of 3∼8 mice per group. ^*^ *P* < 0.05 versus mice fed a chow diet.

A complete ligation of the left common carotid artery of Apoe^-/-^ mice caused no mortality within the 4-week observation period. Lesion formation in the ligated artery was evaluated at multiple time points under both chow and Western diet conditions. Intimal lesions appeared at 1 week after ligation on the Western diet and 2 weeks after ligation on the chow diet (Figure 2). With an increasing post-ligation duration, lesion sizes enlarged in both chow and Western diet groups. At 4 weeks after ligation, the average intimal lesion area of ligated carotid arteries was approximately 4-fold larger in the Western diet group than that in the chow diet group (112,987 ± 11,395 vs. 29,539 ± 7,001 μm^2^/section; n=5 and 8, respectively). The difference was highly statistically significant (*P* = 0.00047).

The medial area of ligated carotid arteries was generally larger than the area of contralateral right carotid arteries at 1, 2 and 4 weeks after ligation, regardless of diet (Figure 2). At 4 weeks, the medial area of ligated carotid arteries had doubled the area of contralateral arteries in both diet groups. The areas within the external or internal elastic lamina in the ligated carotid arteries were comparable between the two diet groups at 1 and 2 weeks after ligation. However, at 4 weeks, these areas were approximately twice larger in the Western diet group than the chow diet group (*P* = 0.00077 and 0.001, respectively).

**Figure 3:**
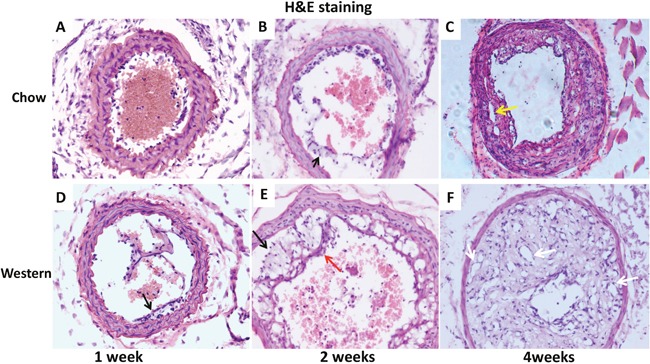
Representative photographs of cross-sections of ligated common carotid arteries from Apoe^-/-^ mice 1, 2 and 4 weeks after ligation The top row shows cross sections of the vessels from mice fed a chow diet **(A, B, C)**, and the bottom row shows cross sections from mice fed a Western diet **(D, E, F)**. Black arrows point at foam cells, red arrow at fibrous cap, white arrows at neovessels, and yellow arrow at cholesterol cleft. Sections were stained with the standard hematoxylin-eosin (H&E) method. Original magnification: ×20.

Histologically, intimal lesions at 1 week after ligation on the Western diet and 2 weeks on the chow diet possessed the nature of fatty streaks comprised primarily of foam cells (Figure 3B, 3D and 3E). These cells were larger than other cells in the lesions with a lightly stained cytoplasm and a foamy morphology on H&E sections. At 2 weeks after ligation on the Western diet, the intimal lesions were typical of fibrous plaques containing numerous foam cells covered by a fibrous cap (Figure 3E). At 4 weeks, intimal lesions of both diet groups progressed to more advanced stages, containing neovessels and substantially narrowing the arterial lumen (Figure 3C and 3F).

**Figure 4:**
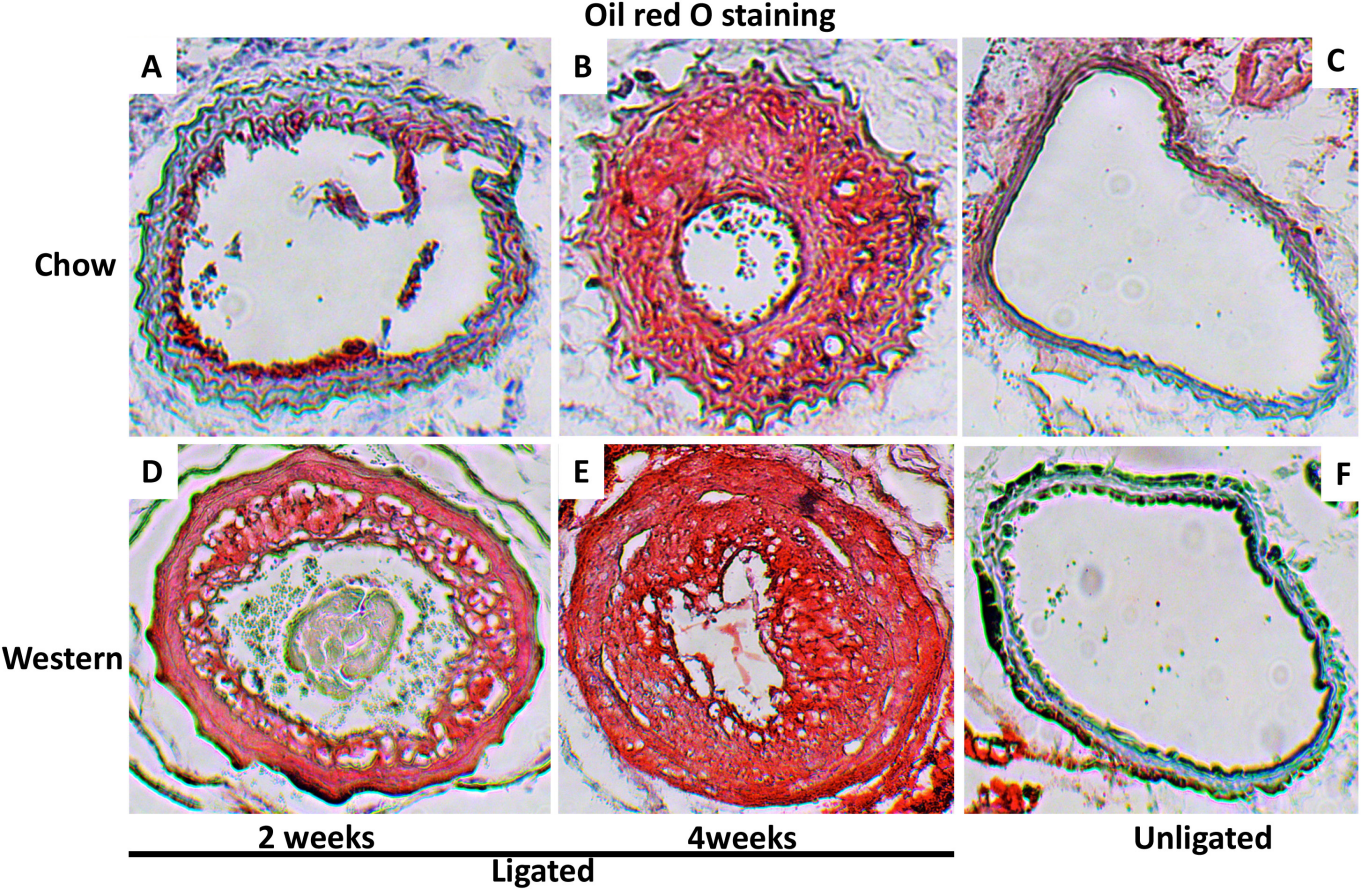
Cross-sections of ligated common carotid arteries **(A, B, D, E)** and unligated right carotid arteries **(C, F)** stained with oil red O. Note intense staining of intimal lesions in the ligated arteries of Apoe^-/-^ mice fed either chow or Western diet and in the medial layer of ligated arteries in mice fed the Western diet. There were no lesions in the unligated carotid arteries. Original magnification: ×10.

The intimal lesions of ligated arteries from either diet group intensely stained with oil red O, suggesting richness in lipids (Figure 4). The medial layer of ligated arteries showed little staining at 2 weeks after ligation (Figure 4A) but grossly stained at 4 weeks on sections from mice fed the chow diet (Figure 4B). The medial layer intensely stained with oil red O at both time points on sections from mice fed the Western diet (Figure 4D and 4E). The contralateral right common carotid artery developed no lesions in either diet group. The medial layer of the artery showed little staining (Figure 4C and 4F).

### Cytological changes during intimal lesion formation

**Figure 5:**
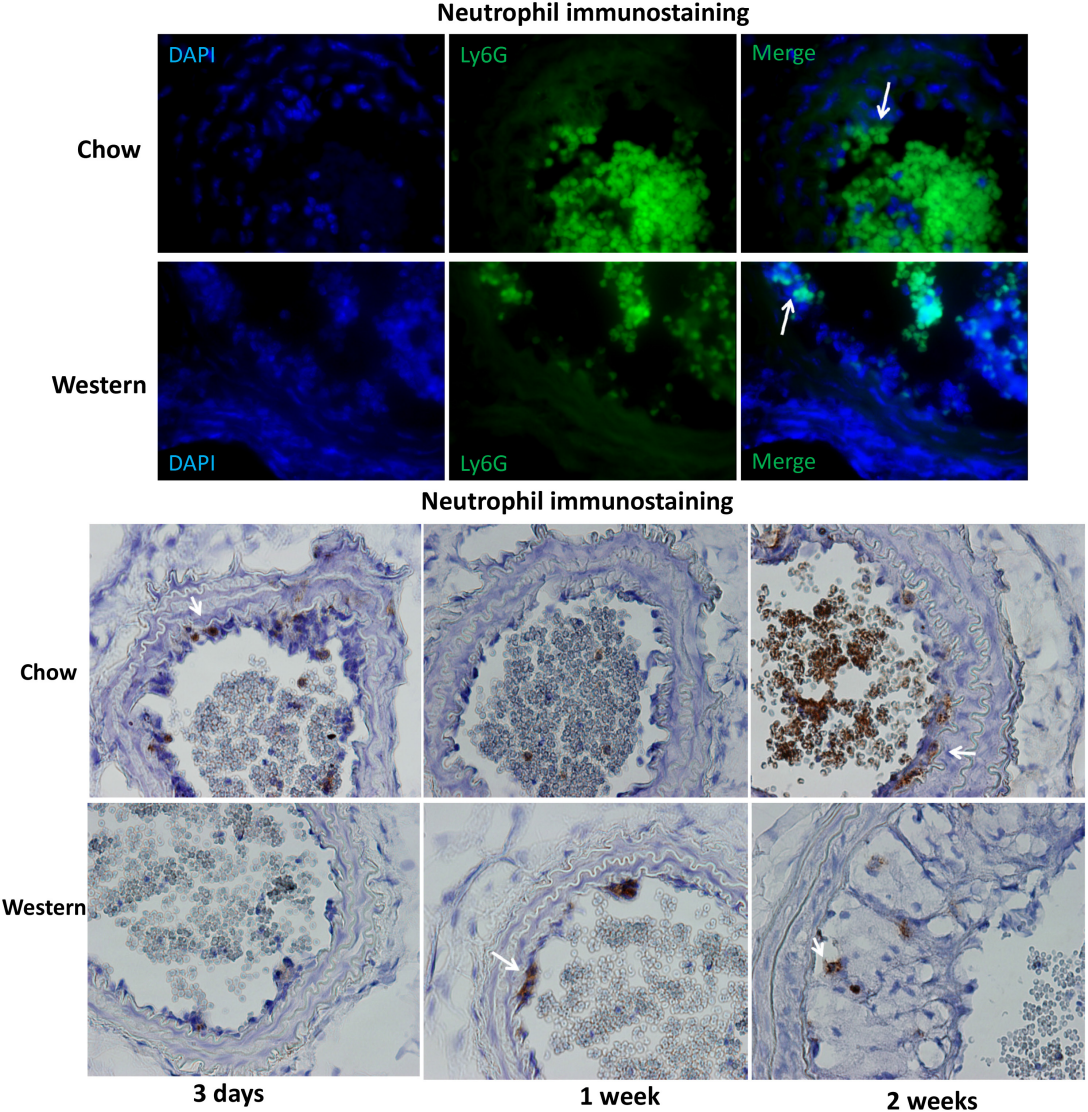
Immunocytochemical detection of neutrophils in the ligated carotid artery of Apoe-/- mice fed a chow or Western diet **(A)** sections were stained with FITC conjugated anti-neutrophil antibody (green) and DAPI (blue). Arrows point to neutrophils attached to the endothelium. **(B)** section stained with the standard Avidin-Biotin Complex (ABC) method using a biotinated anti-neutrophil antibody. Arrows point to stained neutrophils.

One day after ligation, neutrophils were found to be attached to the endothelium of carotid arteries (Figure 5A). Neutrophil infiltration was also observed in growing intimal lesions and at the interface with the medial layer (Figure 5B). This infiltration was observed in ligated arteries of both diet groups.

**Figure 6:**
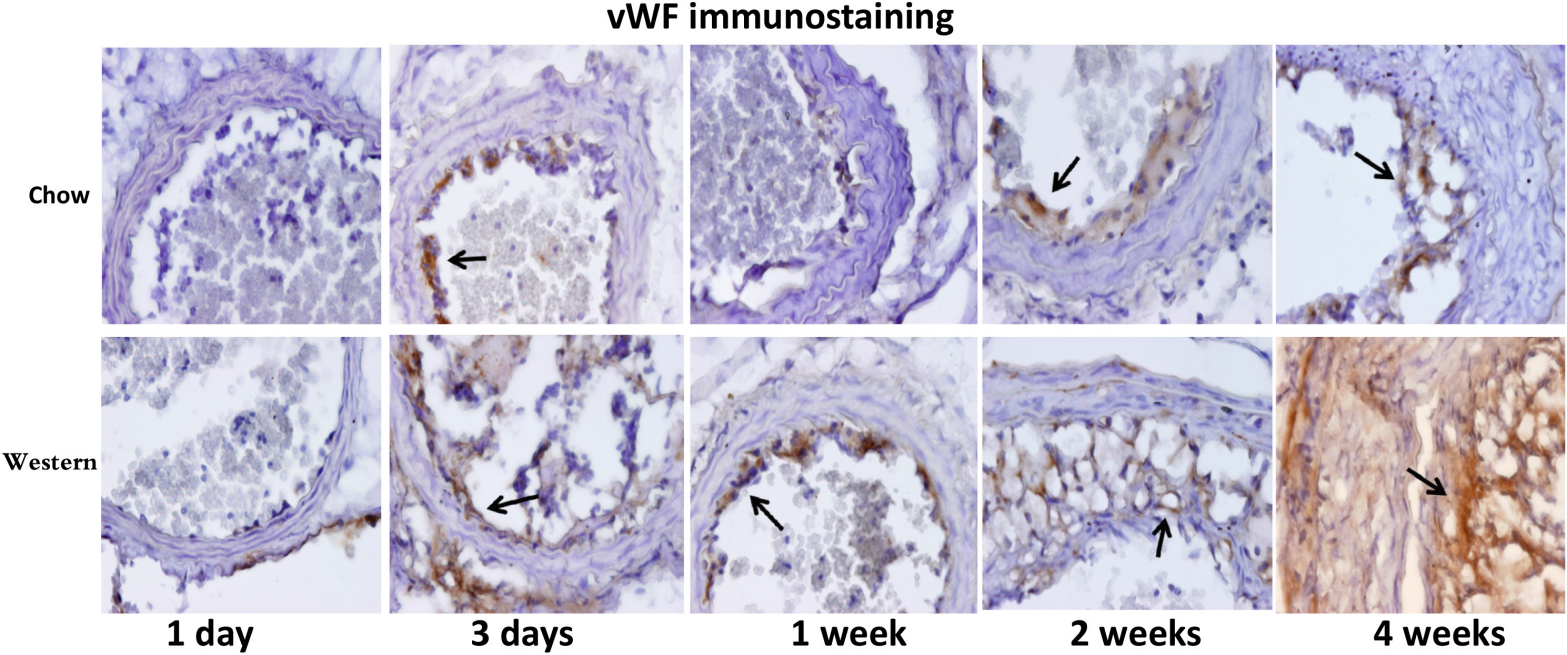
Immunohistochemical staining for von Willebrand Factor (VWF) in the ligated carotid arteries of Apoe^-/-^ mice fed a chow diet (top row) or a Western diet (bottom row) 1 day, 3 days, 1, 2 and 4 weeks after ligation Arrows point to stained areas.

Immunoreactivity to vWF was not obvious in the ligated carotid artery at 1 day after ligation, but a dense stain was observed in regions of the intima or intimal lesions at day 3 and all subsequent time points in both diet groups (Figure 6). Four weeks after ligation, strong immunoreactivity to vWF was observed not only in intimal lesions but also throughout the medial wall of ligated arteries in the Western diet group.

**Figure 7:**
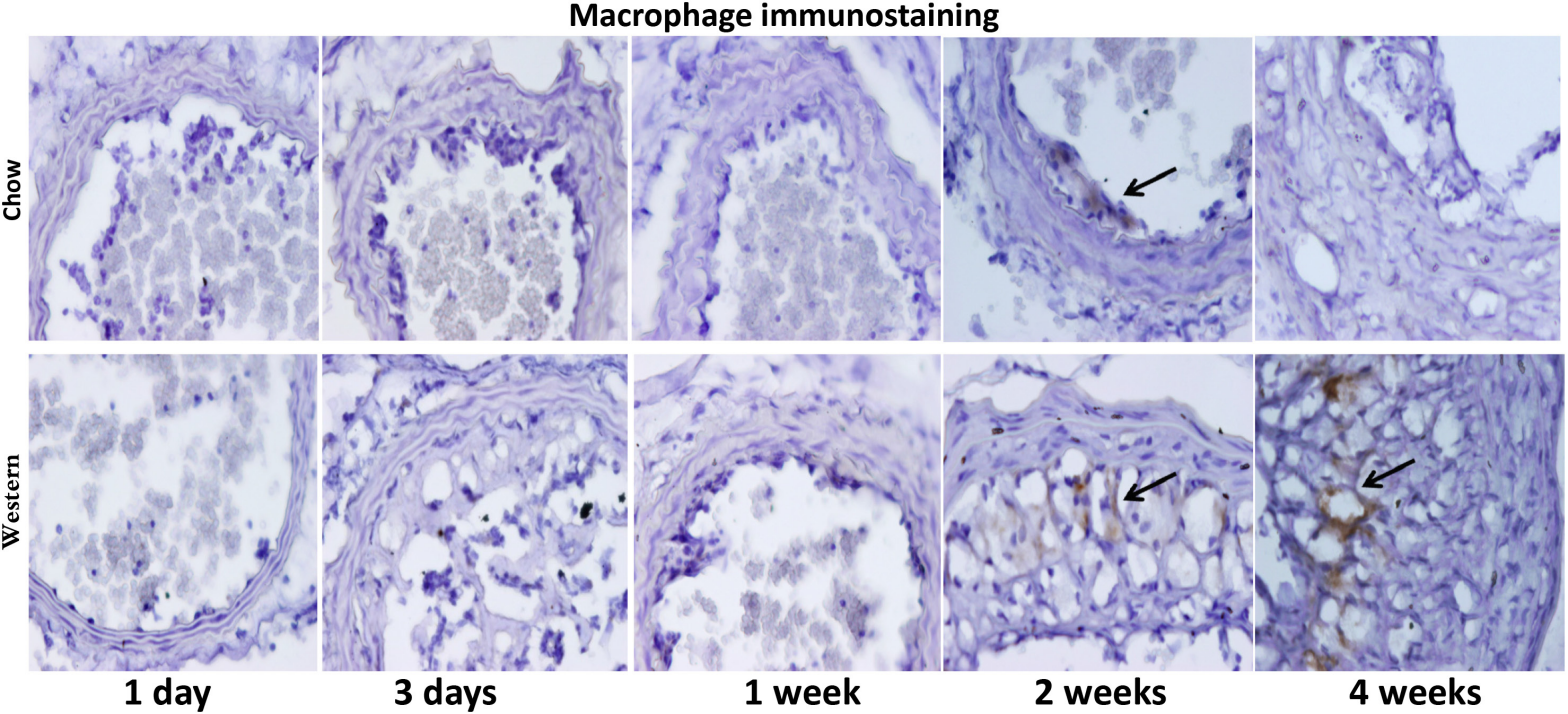
Immunocytochemical detection of macrophages in ligated carotid arteries of Apoe^-/-^ mice with different post-ligation durations The top row shows sections from mice fed a chow diet, and the bottom row shows sections from mice fed a Western diet. Arrows point to stained areas.

No macrophages were detectable in the ligated carotid arteries of either diet group at earlier time points (Figure 7). Macrophage staining in intimal lesions was obvious at later time points, such as 2 and 4 weeks after ligation, especially in mice fed the Western diet.

**Figure 8:**
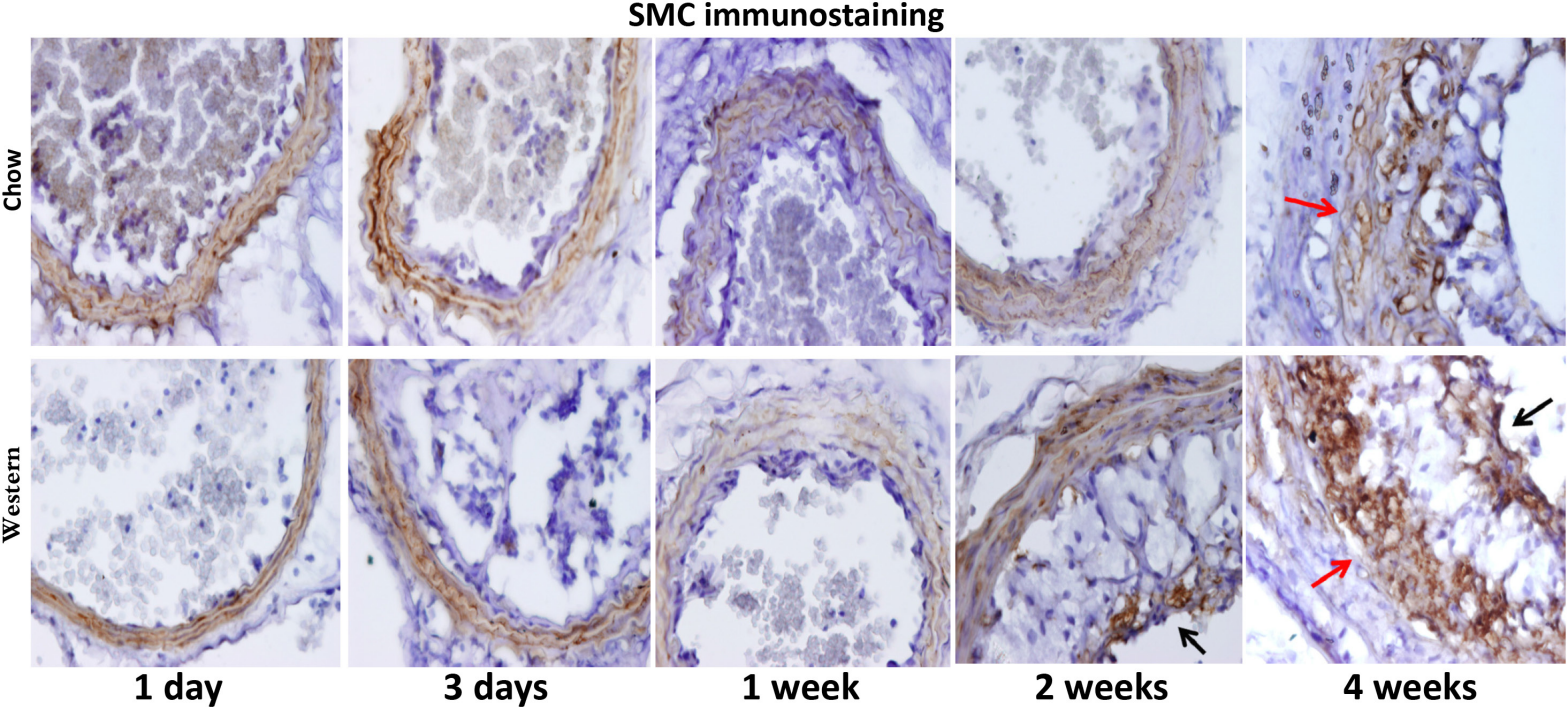
Immunohistochemical staining for α-smooth muscle actin in the ligated carotid artery of Apoe^-/-^ mice fed a chow diet (top row) or a Western diet (bottom row) with different post-ligation durations Note the disorganization of the medial layer 4 weeks after ligation (denoted by red arrow). Black arrows point to stained areas in the cap.

Immunoreactivity to α-smooth muscle actin was observed in the medial layer of arterial walls and fibrous caps covering intimal lesions (Figure 8). The medial layer at 4 weeks after ligation appeared visiblly disorganized and thickened in both diet groups.

**Figure 9:**
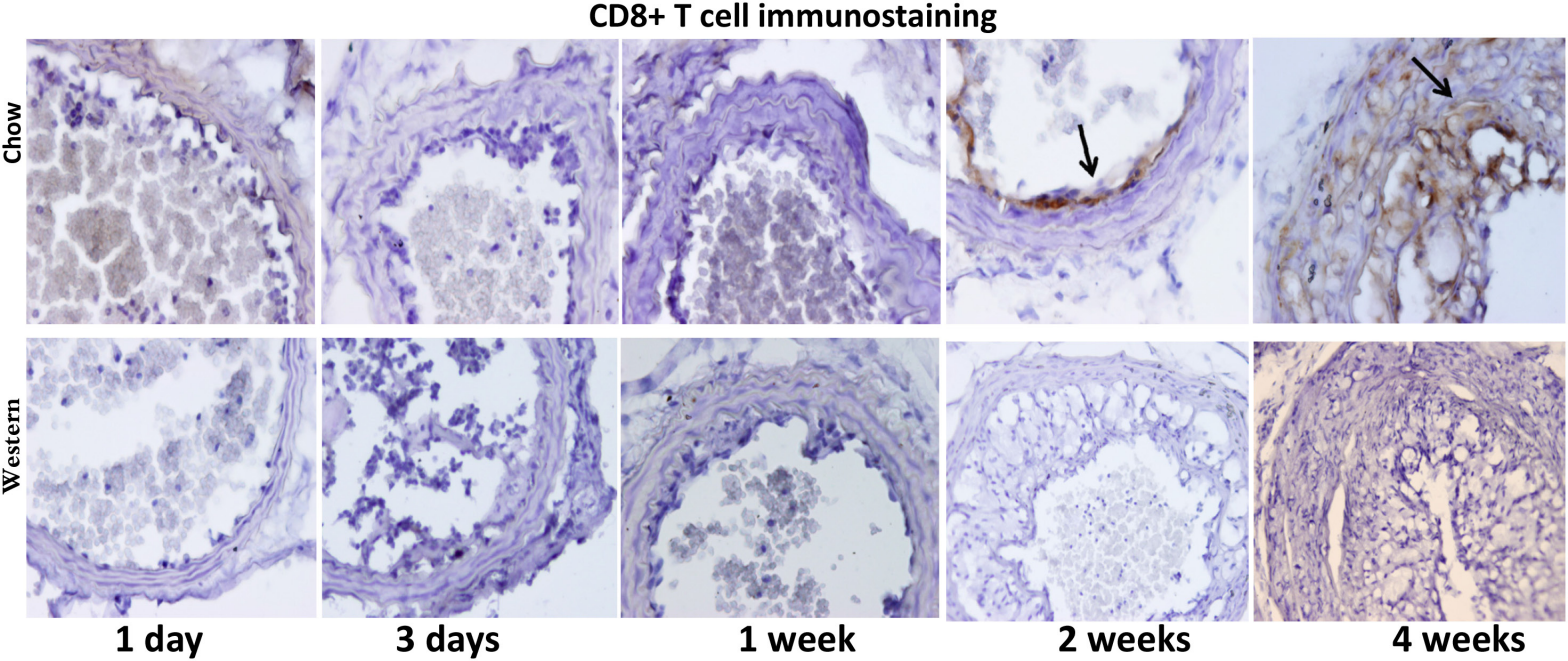
Immunohistochemical staining for CD8 T cells in ligated carotid arteries of Apoe^-/-^ mice 1 day, 3 days, 1, 2 and 4 weeks after ligation The top row shows staining in cross sections from mice fed a chow diet, and the bottom row shows staining in sections from mice fed a Western diet. Note the presence of CD8+ cells in intimal lesions of mice fed a chow diet (denoted by arrow) but not a Western diet.

T lymphocytes were identified with antibodies targeting CD4 and CD8 antigens. Immunoreactivity to the CD8 antigen was observed in intimal lesions and adjacent medial walls in the chow diet group (Figure 9). In contrast, CD8+ T cells were not detectable in the Western diet group. Immunostaining with an antibody directed against CD4 antigen revealed no signal, indicating the absence of CD4+ T cells in the lesions (data not shown).

## DISCUSSION

Partial ligation leads to rapid development of atherosclerosis in the common carotid artery of Apoe^-/-^ mice due to disturbed blood flow [7], but it was unknown what would happen if blood flow was completely blocked. In this study, we characterized intimal lesion formation in completely ligated common carotid arteries of Apoe^-/-^ mice when fed both a chow diet and a Western diet. We found that Apoe^-/-^ mice developed the entire spectrum of atherosclerotic lesions in the ligated artery in an accelerated fashion. Both male and female mice were included in this study as previous studies showed that sex made no difference in intimal lesion formation in ligated common carotid artery of either wild-type B6 mice [11] or Apoe^-/-^ mice [7]. The intimal lesions formed in the ligated carotid artery of Apoe^-/-^ mice bear the histological features of atherosclerotic lesions that develop spontaneously at such sites as arterial branches and curves. Foam cells, the hall mark of atherosclerotic lesions, were observed in the ligated vessels of Apoe^-/-^ mice fed either diet. Both H&E staining and immunocytochemical analysis demonstrated the abundance of lipid-laden, macrophage-derived foam cells in the intimal lesions, especially in mice fed the Western diet. On H&E sections, foam cells are easily recognizeable due to their foamy morphology. Fibrous cap, cholesterol cleft, and neovessels of atherosclerotic plques are also identifiable. Our previous studies demonstrate the reliability of H&E staining in identification of lipid-laden foam cells and neovessels [12][13][14]. Initially, the lesions contained no smooth muscle cells. As they progressed, smooth muscle cells appeared, most of which were located in fibrous caps covering the foam cell-rich areas. As the lesions continued to progress, neocapillaries appeared. Intraplaque neovascularization is considered a major feature of an advanced atherosclerotic lesion [15]. In this study, H&E staining clearly revealed the presence of intraplaque vascular structures. The strong immunoreactivity to the von Willebrand factor (vWF) in lesion areas also suggests the abundance of endothelial cells in the plaques. It is noteworthy that neovascularization was also found in the medial arterial wall underlying the advanced lesions, which probably contributed to the disorientation of smoth muscle cells and the strong immunoreactivity to vWF in the medial layer (Figures 6 and 8). In the advanced stage, the lesions continued to increase in size and increasingly occluded the lumen of ligated arteries.

A previous study reported that partial ligation of the common carotid artery resulted in rapidly developed atherosclerotic lesions in Apoe^-/-^ mice fed a high fat diet [7]. The study has attributed the accelerated atherogenesis to disturbed blood flow. In the present study, we completely ligated the common carotid artery in its distal end and still observed rapidly progressed atherosclerosis in the vessel under a similar dietary condition. Complete ligation in the distal end near the bifurcation caused blood stasis and flow cessation in the common carotid artery although the vessel still experienced arterial blood pressure and pulsation. Thus, the current finding suggests that the shear stress from blood flow is not crucial to the development of atherosclerosis.

Intimal lesion formation in a ligated carotid artery is often limited in most wild-type mouse strains, including B6 mice [6]. In contrast, moderate to significantly sized intimal lesions are observed in B6-Apoe^-/-^ mice, especially when fed a Western diet. The absence of Apoe and the resultant hyperlipidemia should be responsible for enhanced lesion formation in B6-Apoe^-/-^ mice. Indeed, Apoe has been shown to inhibit and hyperlipidemia to promote smooth muscle cell proliferation and vascular remodeling [16][13].

Intense staining for vWF in the intimal region of ligated arteries was observed as early as 3 days after ligation and during the latter observation period. The early vWF stain was probably resultant from increased endothelial secretion as well as the platelet deposition on the surface of the endothelium. Upon adhesion, platelets are activated and secrete pro-inflammatory cytokines and chemoattractants, which promote leukocyte binding to inflamed or atherosclerotic endothelium [17]. Platelets as well as platelet-covered leukocytes have been found to line the endothelium of ligated carotid artery within days [18]. The strong vWF stain was observed in the intimal lesions of mice fed the Western diet. This is in agreement with what was observed in rabbits where high-fat diet induces vWF production and enhances platelet adhesion to subendothelial plaque regions [19]. The enhanced vWF expression in the lesions could partially be attributable to the synthesis and secretion by intraplaque neovessels.

The present study showed an early interaction of neutrophils with endothelial cells in the ligated carotid artery. Neutrophil adherence to endothelial cells was observed one day after ligation, the earliest time point examined. Activated neutrophils release superoxide and pro-inflammatory molecules at the sites of adhesion that promote the recruitment of monocytes and alter endothelial cell properties [20]. A recent study shows that in hypercholesterolemia-induced neutrophilia, cholesterol crystals trigger neutrophils to release neutrophil extracellular traps, which prime macrophages for cytokine release and activate T helper 17 cells and consequently amplify immune cell recruitment in atherosclerotic plaques [21].

An interesting finding of this study is that CD8+ T cells were only observed in the intimal lesions of mice fed the chow diet but not those fed the Western diet. The oxysterol metabolite, 27-hydroxycholesterol, from high fat diet suppresses the recruitment of CD8+ T lymphocytes to peripheral tissues [22]. We previously found that high fat diet induces a chronic inflammatory status with elevated production of proinflammary cytokines in mice [23][24][13][25]. CD8+ T cells promote reendothelialization and inhibit neointima formation following arterial injury in mice [26]. Clinical studies indicate that the neutrophil to lymphocyte ratio reflects the severity of diseases with an inflammatory component and predicts the risk of developing in-stent restenosis and major adverse cardiac events [27][28]. Thus, there is a possibility that the Western diet promotes intimal lesion formation in the ligated carotid artery through action on CD8+ T cells.

On the Western diet, Apoe^-/-^ mice showed a dramatic increase in total cholesterol levels, primarily due to elevations in non-HDL cholesterol, and exhibited accelerated atherosclerotic lesion formation. The lesions in Apoe^-/-^ mice fed the Western diet occurred earlier and were more advanced compared to those in chow fed mice. Besides the effect on cholesterol homeostasis, high fat diet induces a chronic inflammatory status with elevations in circulating proinflammatory molecules, such as VCAM-1, P-selection and MCP-1 [24][25]. Thus, the Western diet accelerated the atherogenic process also through action on inflammation.

An intriguing finding in the present study is that the intimal lesions contained numerous neovessels, especially in mice fed the Western diet. Neoangiogenesis is a histological feature of advanced atherosclerosis in humans [29], but it is rarely seen in animal models, including Apoe^-/-^ mice [30]. Hypoxia has been observed in atherosclerotic plaques [31][32] and considered a major factor behind neoangiogenesis. The rapid progression of intimal lesions and the static blood flow following carotid ligation undoubtedly have aggravated hypoxia in the lesions and consequently accelerated neovessel formation.

In wild-type mouse strains, ligation of the carotid artery causes constriction or shrinkage of the vessel diameter [5][6]. In contrast, such alterations were not found in Apoe^-/-^ mice fed either a chow or Western diet; rather these mice showed an increase in the areas within the internal and external elastic laminae 4 weeks after ligation. This finding is consistent with the vascular remodeling observed in animal models of atherosclerosis and in human coronary arteries with plaques [33][34][35]. Smooth muscle cells are the main component of intimal lesions in wild-type mice [5], while in Apoe^-/-^ mice the intimal lesions contain numerous macrophages. Smooth muscle cells and the extracellular matrix produced by the cells restricts vessel distension [36]. In contrast, macrophages produce MMP-9, MMP-12, and other enzymes that degrade extracellular matrix [37]. The degradation of the extracellular matrix is not only toward to the luminal side of the plaque but also toward the abluminal layer, thus weakening the vessel wall [37]. Moreover, a higher macrophage count and lipid content has shown significant associations with positive remodeling in human coronary artery with plaques [38][39].

In summary, the present study demonstrates that complete carotid ligation results in accelerated atherosclerosis, which rapidly progresses from fatty streak to fibrous lesion to advanced lesion, in Apoe^-/-^ mice. This forms a striking contrast with the slow growth of atherosclerotic lesions in unligated carotid artery of Apoe^-/-^ mice [40]. This model should be a valuable tool for studying the pathogenesis and therapeutic interventions of atherosclerosis.

## MATERIALS AND METHODS

### Mice

B6-Apoe^-/-^ mice purchased from the Jackson Laboratory were bred to generate mice used for the present study. Mice of both sexes were weaned at 3 weeks of age onto a standard chow diet. One group of mice were maintained on a rodent chow diet throughout the entire experimental period. And the other group was fed a Western diet containing 21% fat, 48.5% carbohydrate, 17% protein, and 0.2% cholesterol (by weight) (TD 88137, Envigo), starting 1 week before surgery and being maintained on the diet thereafter. All procedures were carried out under the current NIH guidelines and approved by the Institutional Animal Care and Use Committee.

### Surgical procedure

Ligation of the left common carotid artery was performed as previously described [14]. Briefly, mice aged 4∼8 weeks were anesthetized by intramuscular injection with ketamine (80 mg/kg body weight; Ketaset, Aveco Inc.) and xylazine (8 mg/kg; AnaSed, Lloyd Laboratories). The left common carotid artery was dissected under a microscope and ligated near its bifurcation to completely block blood flow. The skin incision was then closed with a surgical glue (VETCLOSETM, Henry Schein Animal Health). Animals were euthanized 1 day, 3 days, and 1, 2, and 4 weeks after surgery. 3 or more mice were analyzed at each time point for each diet group.

### Tissue preparation and lesion quantification

Mice were euthanized with prolonged exposure to isofluorane inhalation. The vasculature was perfused with 4% paraformaldehyde via the left ventricle of the heart. The neck was dissected and further fixed in the same solution for >24 h. After fixation, the front soft tissues of the neck encompassing the left and right common carotid were dissected out, embedded in OCT compound (Tissue-Tek, Miles Inc), and cross-sectioned in 10-μm thickness. Serial sections were collected, starting from disappearance of the ligation suture, and mounted on poly-D-lysine-coated slides with 6∼8 sections per slide. Approximately 400 sections were collected for each mouse. Three evenly spaced slides were chosen for hematoxylin and eosin (H&E) staining. One section on each stained slide was subject to morphometric measurents of the ligated left common carotid artery and the contralateral right common carotid artery using Zeiss AxioVision 4.8 software. Luminal area and areas encircled by the internal and external elastic laminae were measured. Lesion area was calculated by subtracting the luminal area from the area surrounded by the internal elastic lamina, and the medial area of the arterial wall was calculated as the difference between the areas encircled by the external and internal elastic laminae. Measurements made from 3 separate slides were averaged for each vessel and this average was used for statistical analysis. For visualization of neutral lipid, selected sections were stained with oil red O and hematoxylin, counterstained with fast green [24].

### Immunohistochemical analysis

Immunohischemical staining for smooth muscle cell α-actin, von Willebrand factor (vWF), and leukocyte specific markers was performed on frozen sections of the carotid arteries using the following primary antibodies: Mouse anti-human α-smooth muscle actin IgG (Dako Corp.); rat anti-mouse macrophage/monocyte IgG, clone MOMA-2 (Serotec); rabbit anti-human von Willebrand factor (vWF) (Sigma); rat anti-mouse CD4 antibody (Millipore); rabbit anti-mouse CD8 IgG (Cell Signaling); rat anti-mouse Ly-6G antibody (eBioscience); and FITC anti-mouse Ly-6G IgG (BD Biosciences). Subsequent incubations with biotinylated secondary antibody and VECTASTAIN Elite ABC HRP Kit (Vector Laboratories) were performed as previously described [41]. Sections stained with fluorescent-labeled antibody or DAPI fluoromount-G (Southern Biotech) were directly visualized with a fluorescence microscope.

### Plasma lipid measurements

Mice were fasted overnight before blood was collected through retro-orbital sinus puncture under isoflurane anesthesia. Plasma total cholesterol, HDL cholesterol, and triglyceride levels were measured using the Thermo DMA (Louisville, CO) cholesterol and triglyceride kits [16]. Non-HDL was calculated as the difference between total and HDL cholesterol levels.

### Statistical analysis

All values were expressed as mean ± SE, with “n” indicating the number of mice. Student's t test was used to determine statistical differences between the two groups for morphometric measurements and plasma parameters. Differences were considered statistically significant at *P* ≤ 0.05.

## SUPPLEMENTARY MATERIALS




