# Supplementary material for: Accelerated atherogenesis in completely ligated common carotid artery of apolipoprotein E-deficient mice
Source: Oncotarget. 2017 Nov 25;8(66):110289–99. doi: 10.18632/oncotarget.22685 (PMC5746382; doi:10.18632/oncotarget.22685)
Supplement: Supplementary file 1 [file oncotarget-08-110289-s001.pdf]

Accelerated atherogenesis in completely ligated common carotid artery of apolipoprotein E-deficient mice

SUPPLEMENTARY MATERIALS

Supplementary Materials

See Supplementary File 1
